# Patient-reported outcomes of symptom burden and quality of life in patients with polycythemia vera and essential thrombocythemia in Korea: A cross-sectional study

**DOI:** 10.1097/MD.0000000000050177

**Published:** 2026-08-28

**Authors:** Yoorin Cho

**Affiliations:** a Department of Nursing, Red Cross College of Nursing, Chung-Ang University, Seoul, Republic of Korea.

**Keywords:** essential thrombocythemia, hematological cancer, polycythemia vera, quality of life

## Abstract

This cross-sectional survey aimed to identify factors affecting quality of life (QoL) in patients with polycythemia vera (PV) and essential thrombocythemia (ET). Participants comprised 189 patients with PV or ET receiving outpatient treatment in Seoul. Data were collected using a structured questionnaire and analyzed using linear multiple regression analysis. Factors affecting the QoL in patients with PV or ET, including overall QoL, functional scales, and symptom experience, were fatigue, disease duration, numbness/tingling, bone pain, fever, weight loss, abdominal discomfort, educational level, and female sex. Assessment of QoL and symptom burden in patients with PV and ET indicated that fatigue, disease duration, numbness/tingling, bone pain, fever, weight loss, abdominal discomfort, educational level, and female sex significantly influence QoL, highlighting the importance of active management and continuous monitoring of patient symptoms. Future research should evaluate the impact of comorbid conditions on symptom burden and QoL.

## 1. Introduction

Polycythemia vera (PV) and essential thrombocythemia (ET) are the most common Philadelphia chromosome-negative myeloproliferative neoplasms,^[1]^ characterized by the overproduction of red blood cells, megakaryocytes, and granulocytes, splenomegaly, and thrombotic events. Disease progression can lead to myelofibrosis, myelodysplastic syndrome, or acute myeloid leukemia. The average age at diagnosis for patients with PV and ET is 60 years,^[2]^ with incidence rates in the United States of 1.56 and 1.55 per 1,00,000 people, respectively,^[3]^ and in Korea of 0.31 and 0.64 per 1,00,000 people, respectively.^[4]^ Current risk stratification in PV and ET focuses on predicting the likelihood of recurrent thrombosis. In PV, patients older than 60 years or with a history of thrombosis are classified as high-risk due to an increased risk of future thrombotic events.^[2,5]^ ET is classified into 4 risk groups according to the Revised International Prognostic Score of Thrombosis in Essential Thrombocythemia (R-IPSET). Very low risk includes patients ≤60 years old without a history of thrombosis and without a JAK2 mutation; low risk includes those with only a JAK2 mutation; intermediate risk includes patients>60 years old without a history of thrombosis and without a JAK2 mutation; and high risk includes patients with a history of thrombosis or those >60 years old with a JAK2 mutation.^[2,6]^ Although PV is associated with higher morbidity and mortality,^[7]^ ET treatment contributes mainly to symptom control rather than survival improvement.^[3]^ Major symptoms of PV include pruritus, fatigue, reduced concentration, splenomegaly, bleeding, and thrombotic complications,^[8]^ whereas ET symptoms range from asymptomatic presentations to thrombosis, elevated platelet counts, night sweats, pruritus, and extreme fatigue.^[9,10]^ Treatments for PV and ET aim to reduce thrombotic risk with low-dose antiplatelet therapy while alleviating symptoms using medications such as hydroxyurea, interferon-alpha, busulfan, and ruxolitinib.^[2]^ Allogeneic hematopoietic stem cell transplantation can reduce symptom burden but is limited to selected patients due to severe adverse effects and low survival rates.^[2,11,12]^ Despite these treatment approaches aimed at improving various symptoms and enhancing quality of life (QoL), patients may experience decreased physical strength, reduced physical activity, social isolation, impaired role functioning, and financial difficulties over prolonged treatment periods, leading to reduced QoL. In particular, the lack of established optimal therapies results in uncertainty regarding treatment efficacy and challenges in managing QoL.^[13-15]^

Furthermore, the average age at diagnosis of 60 years often leads patients to misinterpret disease-related symptoms as normal aging or worsening of comorbid conditions, resulting in missed opportunities for timely treatment.^[16]^ Consequently, patients may present to hospitals with severe systemic symptoms and complications. Women and older adults, in particular, have been shown to have lower QoL.^[17]^ A limited understanding of the disease among patients with PV and ET, combined with the infrequent exposure of healthcare providers to these conditions, can create challenges in the treatment process for both patients and clinicians, ultimately negatively affecting patient QoL.^[18-20]^ Healthcare providers caring for patients with PV and ET assess their current condition through risk stratification and evaluate QoL based on the severity of symptom burden.^[18]^ Most previous research has focused primarily on treatment outcomes; therefore, studies assessing the impact of symptom burden are needed to improve patients’ QoL.

International studies have investigated symptom burden and QoL by grouping myelofibrosis (MF) with PV and ET as myeloproliferative neoplasms.^[15,18,20-25]^ However, MF progresses rapidly, has a shorter survival period, and requires aggressive treatment, whereas PV and ET show chronic disease-like progression and longer life expectancy compared to other hematologic malignancies. Additionally, PV and ET treatment primarily focuses on thrombosis prevention.^[26]^ Therefore, this study aimed to examine symptom burden and QoL exclusively in patients with PV and ET and to identify factors influencing QoL, providing foundational data for developing strategies to enhance QoL.

## 2. Design

This study was a cross-sectional survey aimed at assessing symptom burden and QoL in patients with PV and ET and identifying factors influencing QoL.

## 3. Participants

Participants were patients diagnosed with PV or ET receiving treatment at Seoul St. Mary’s Hospital in Seoul, South Korea. Inclusion criteria were: age ≥20 years; ability to understand and respond to the questionnaire; voluntary consent to participate in the study; and more than 1 month since the initial diagnosis. Exclusion criteria were: a history of cancer other than PV or ET, and an Eastern Cooperative Oncology Group (ECOG) performance status ≥3. The rationale for including patients at least 1 month post-diagnosis was to avoid the emotional distress immediately following diagnosis, to reflect early treatment experiences, and to allow patients to evaluate their symptoms and QoL more stably.

Using G*Power 3.1.9.4, with a significance level of 0.05, a medium effect size of 0.15, and power of 0.95, the required sample size for the linear regression analysis was calculated as 172 participants. A total of 189 participants completed the questionnaire.

## 4. Materials and methods

### 4.1. Myeloproliferative Neoplasm Symptom Assessment

Symptom burden was assessed using the Korean version of the Myeloproliferative Neoplasm Symptom Assessment Form Total Symptom Score (MPN-SAF TSS), provided by MD Anderson, with verified reliability and validity. The MPN-SAF TSS evaluates 10 symptoms associated with myelofibrosis: fatigue, early satiety, abdominal discomfort, bone pain, pruritus, night sweats, weight loss, numbness/tingling, fever, and impaired concentration. Each symptom is rated from 0 (absent) to 10 (severe), with scores converted to a total of 100 points. Higher scores indicate greater symptom severity.^[27]^

### 4.2. Quality of life

Health-related QoL was assessed using the European Organization for Research and Treatment of Cancer Quality of Life Questionnaire-Core 30 (EORTC QLQ-C30, version 3.0). The EORTC QLQ-C30 consists of 30 items across 3 subscales: global health status (2 items), functional domains (15 items), and symptom domains (13 items). Responses for global health status are rated on a 7-point scale, and those for functional and symptom domains on a 4-point scale. Scores were converted to a 0–100 scale according to the EORTC QLQ-C30 scoring manual, with lower scores in global health status and functional domains and higher scores in symptom domains indicating poorer QoL.^[28]^

### 4.3. Data collection

The study protocol was approved by the Institutional Review Board of the Catholic University of Korea, Seoul St. Mary’s Hospital (No. IRB:KC21QISI0929). Eligible participants were recruited from the outpatient clinic of St. Mary’s Hospital. The researcher had no involvement in the participants’ clinical care and was not part of their treatment team. Before the survey, the researcher explained the study purpose and procedures to each participant and obtained written informed consent. Participants were told that their data would be used only for research and that they could withdraw at any time without disadvantage. Participants completed the questionnaire themselves, taking approximately 10 minutes on average. Between January and August 2023, 189 patients were surveyed. Diagnosis dates, laboratory results, and other relevant information were confirmed through electronic medical records. As diagnosis dates and laboratory results were verified through electronic medical records and questionnaires were completed on-site, the dataset contained no missing values.

### 4.4. Risk group classification

Thrombosis risk was classified separately for PV and ET according to established criteria. Patients with PV were categorized as low risk (age ≤60 years and no history of thrombosis) or high risk (age >60 years or a history of thrombosis). Patients with ET were initially classified into 4 risk categories according to the revised International Prognostic Score for Essential Thrombocythemia (R-IPSET), namely very low, low, intermediate, and high risk. To facilitate comparison with the 2-tier risk classification used for PV, the very low- and low-risk categories were combined into a low-risk group, whereas the intermediate- and high-risk categories were combined into a high-risk group.

### 4.5. Data analysis

Collected data were analyzed using SPSS (Statistical Package for the Social Sciences) version 27.0 (IBM Corp., Armonk). Descriptive statistics, including frequencies, percentages, means, and standard deviations, were used to summarize participants’ general characteristics, symptom burden, and QoL levels. Factors influencing QoL were examined using multiple linear stepwise regression analysis after checking for multicollinearity.

## 5. Result

### 5.1. Clinical and demographic characteristics of patients

The mean age of the participants was 57.5 years, with the largest group being those aged ≥61 years (n = 92, 48.7%). Female participants numbered 95 (50.3%). Most were married (n = 158, 83.6%), 95 (50.3%) had a college degree or higher, 101 (53.4%) were unemployed, and 95 (50.3%) reported no religious affiliation. A monthly household income of ≥3 million KRW was reported by 111 participants (58.7%). The diagnoses included PV in 87 patients (46.0%) and ET in 102 patients (54.0%), with the majority having a disease duration of 1–5 years post-diagnosis (n = 86, 45.5%). Aspirin was the most commonly used treatment (n = 169, 89.4%; Table 1).

**Table 1:** Clinical and demographic characteristics of participants.

	N = 189
Characteristics	n(%)/ M ± SD (range)
Age (yr)	57.5 ± 15.4 (22–91)
≤39	25 (13.2)
40–60	72 (38.1)
≥61	92 (48.7)
Sex	
Male	94 (49.7)
Female	95 (50.3)
Marital status	
Married	158 (83.6)
Unmarried	31 (16.4)
Education	
≤Middle school	32 (16.9)
High school	62 (32.8)
≥College	95 (50.3)
Employment	
Employed	88 (46.6)
Unemployed	101 (53.4)
Religious	
Yes	94 (49.7)
No	95 (50.3)
Monthly income	
Yes	31 (16.4)
No	158 (83.6)
Monthly income (Won)	
<300	78 (41.3)
≥300	111 (58.7)
Diagnosis	
Polycythemia vera	87 (46.0)
Essential thrombocythemia	102 (54.0)
Blood test result*	
Hematocrit < 45%	111 (58.7)
WBC < 10 × 10^9^/L	115 (60.8)
Platelets < 400 × 10^9^/L	64 (33.9)
Presence of mutation^*^	
JAK2	126 (66.7)
CALR	29 (15.3)
MPL	2 (1.1)
Triple negative	33 (17.5)
Time since diagnosis (yr)	
<1	44 (23.3)
1–5	86 (45.5)
>5	59 (31.2)
Treatment*	
Aspirin	169 (89.4)
Hydroxyurea	133 (70.4)
Anagrelide	86 (45.5)
Interferon	12 (6.4)
Phlebotomy	76 (40.2)
Caregiver	
Spouse	91 (48.1)
Nobody	51 (27.0)
Children	28 (14.8)
Others†	19 (10.1)

CALR = calreticulin, JAK2 = mutations in Janus kinase 2, MPL = myeloproliferative leukemia virus.

* Multiple response.

† Others = parents, siblings, relatives, social services.

### 5.2. Assessment of myeloproliferative neoplasm symptoms

The mean total symptom scores out of 100 were 22.12 for patients with PV and 20.73 for patients with ET, with 96.6% and 94.0% of patients, respectively. Fatigue was the most common symptom, experienced by 93.3% of PV patients and 93.0% of ET patients, and also showed the highest mean severity score in both groups (PV: 4.09; ET: 4.22, out of 10). In PV, fatigue was followed in frequency by numbness/tingling (71.9%), early satiety and itching (both 67.4%), concentration problems (65.2%), and abdominal discomfort (64.0%). In ET, fatigue was followed by early satiety and numbness/tingling (both 71.0%), concentration problems (63.0%), abdominal discomfort (60.0%), and itching (55.0%).

When further stratified by thrombosis risk within each disease group, the total symptom score did not differ significantly between low- and high-risk patients in either PV (*P* = .443) or ET (*P* = .259). Among individual symptoms, weight loss was significantly more severe in high-risk patients in both PV (*P* = .048) and ET (*P* = .024), while abdominal discomfort was significantly more severe in low-risk than in high-risk ET patients (*P* = .031). No other symptom items differed significantly by risk group in either disease (Table 2).

**Table 2:** Myeloproliferative neoplasm symptoms assessment in patients with polycythemia vera and essential thrombocythemia.

					N = 189			
Variable (range)	Polycythemia vera				Essential thrombocythemia			
	Total (Inc, %)	Low risk (M ± SD)	High risk (M ± SD)	*P*	Total (Inc, %)	Low risk (M ± SD)	High risk (M ± SD)	*P*
Total symptom score (0–100)	22.12 ± 18.53 (96.6)	23.24 ± 18.64	20.98 ± 18.56	.443	20.73 ± 15.18 (94.0)	22.52 ± 15.53	18.79 ± 14.71	.259
Fatigue (0–10)	4.09 ± 2.64 (93.3)	4.49 ± 2.67	3.68 ± 2.59	.160	4.22 ± 2.73 (93.0)	4.58 ± 2.72	3.83 ± 2.72	.172
Early satiety (0–10)	2.78 ± 2.84 (67.4)	3.07 ± 3.15	2.48 ± 2.49	.453	2.71 ± 2.59 (71.0)	3.00 ± 2.60	2.40 ± 2.57	.214
Abdominal discomfort (0–10)	2.46 ± 2.68 (64.0)	2.84 ± 2.69	2.07 ± 2.65	.092	2.15 ± 2.65 (60.0)	2.63 ± 2.80	1.62 ± 2.38	.031
Numbness/tingling (0–10)	3.06 ± 2.87 (71.9)	3.49 ± 3.02	2.61 ± 2.68	.176	3.07 ± 2.92 (71.0)	3.31 ± 2.84	2.81 ± 3.02	.245
Concentration problems (0–10)	2.60 ± 2.78 (65.2)	2.71 ± 2.94	2.48 ± 2.64	.946	2.62 ± 2.91 (63.0)	2.92 ± 3.15	2.29 ± 2.62	.312
Night sweats (0–10)	1.61 ± 2.64 (39.3)	1.58 ± 2.80	1.64 ± 2.50	.445	0.95 ± 1.69 (39.0)	1.08 ± 1.94	0.81 ± 1.38	.705
Itching (0–10)	2.70 ± 3.07 (67.4)	3.11 ± 3.52	2.27 ± 2.49	.617	2.13 ± 2.70 (55.0)	2.54 ± 2.83	1.69 ± 2.51	.097
Bone pain (0–10)	2.04 ± 3.03 (48.3)	1.82 ± 2.97	2.27 ± 3.11	.296	1.89 ± 2.78 (45.0)	1.77 ± 2.58	2.02 ± 3.01	.904
Fever (0–10)	0.43 ± 1.50 (14.6)	0.42 ± 1.67	0.43 ± 1.32	.377	0.10 ± 0.44 (6.0)	0.08 ± 0.33	0.12 ± 0.53	.900
Weight loss (0–10)	0.80 ± 1.73 (28.1)	0.56 ± 1.56	1.05 ± 1.87	.048	0.89 ± 2.12 (21.0)	0.62 ± 2.02	1.19 ± 2.20	.024

*P*-values were obtained using the Mann–Whitney *U* test (low vs high risk within each subtype).

Risk group was defined separately for each disease: for PV, low risk (age ≤ 60 years and no history of thrombosis) versus high risk (age > 60 years or a history of thrombosis); for ET, the 4 R-IPSET categories were collapsed into low risk (very low + low) and high risk (intermediate + high) for comparison with the two-tier PV classification.

ET = essential thrombocythemia, M = mean, MPN-SAF TSS = Myeloproliferative Symptom Assessment Form Total Symptom Score, PV = polycythemia vera, SD = Standard deviation.

### 5.3. Quality of life

The mean global health status/QoL measured by the EORTC QLQ-C30 were 61.06 and 61.10 out of 100 for patients with PV and ET, respectively. The mean functional scale scores were 72.96 and 74.01, respectively, while the mean symptom experience scores were 21.21 and 19.69, respectively. Among symptoms, fatigue was the most severe (35.21 and 34.81 for PV and ET, respectively), followed by insomnia (33.61 and 29.61), dyspnea (24.99 and 21.57), constipation (20.17 and 21.61), and pain (21.56 and 18.38).

Stratified by thrombosis risk, global health status/QoL, functional scale, and symptom scale scores did not differ significantly between low- and high-risk patients within either PV or ET (*P* > .05). At the individual domain level, high-risk ET patients reported significantly worse appetite loss than low-risk ET patients (*P* = .022), whereas emotional functioning was significantly better in high-risk than low-risk ET patients (*P* = .045). No significant risk-group differences were observed among PV patients for any QoL domain (Table 3).

**Table 3:** Quality of life in polycythemia vera and essential thrombocythemia patients.

N = 189								
Variable (range)	Polycythemia vera				Essential thrombocythemia			
	Total (M ± SD)	Low risk (M ± SD)	High risk (M ± SD)	*P*	Total (M ± SD)	Low risk (M ± SD)	High risk (M ± SD)	*P*
Global health status/QoL (0–100)	61.06 ± 22.97	61.84 ± 22.18	60.25 ± 23.98	.881	61.10 ± 23.56	63.25 ± 20.46	58.77 ± 26.54	.545
Functional scales (0–100)	72.96 ± 13.56	73.36 ± 12.50	72.54 ± 14.70	.948	74.01 ± 11.76	74.52 ± 11.47	73.47 ± 12.17	.614
Physical functioning	76.71 ± 23.16	80.40 ± 18.56	72.93 ± 26.77	.222	80.37 ± 18.41	83.44 ± 13.24	77.04 ± 22.40	.353
Role functioning	80.33 ± 27.70	83.73 ± 25.72	76.84 ± 29.48	.185	80.67 ± 27.39	85.92 ± 22.20	74.98 ± 31.33	.065
Emotional functioning	79.70 ± 20.35	78.69 ± 20.12	80.73 ± 20.77	.548	79.55 ± 19.89	76.33 ± 20.09	83.04 ± 19.27	.045
Cognitive functioning	80.99 ± 21.15	80.69 ± 22.39	81.30 ± 20.06	.920	80.60 ± 21.12	80.06 ± 24.70	81.19 ± 16.64	.582
Social functioning	81.07 ± 24.63	78.49 ± 25.51	83.70 ± 23.70	.238	83.98 ± 23.89	84.60 ± 23.74	83.31 ± 24.29	.811
Symptom scales (0–100)	21.21 ± 18.58	20.16 ± 17.61	22.29 ± 19.67	.712	19.69 ± 15.70	18.27 ± 14.64	21.23 ± 16.79	.525
Fatigue	35.21 ± 24.87	36.20 ± 24.67	34.20 ± 25.31	.576	34.81 ± 26.21	37.10 ± 25.40	32.33 ± 27.11	.237
Nausea/vomiting	9.82 ± 17.57	8.96 ± 17.27	10.70 ± 18.03	.504	9.39 ± 15.62	11.92 ± 18.21	6.65 ± 11.79	.173
Pain	21.56 ± 28.65	16.71 ± 25.12	26.52 ± 31.37	.102	18.38 ± 23.64	15.12 ± 19.33	21.92 ± 27.34	.387
Dyspnea	24.99 ± 28.11	23.62 ± 29.84	26.39 ± 26.50	.408	21.57 ± 27.36	19.13 ± 24.09	24.21 ± 30.54	.550
Insomnia	33.61 ± 31.23	33.24 ± 32.61	33.98 ± 30.12	.789	29.61 ± 30.36	24.25 ± 24.81	35.42 ± 34.74	.150
Appetite loss	19.42 ± 28.79	13.27 ± 22.88	25.70 ± 32.86	.063	15.64 ± 26.60	9.60 ± 20.19	22.19 ± 31.04	.022
Constipation	20.17 ± 28.27	22.16 ± 28.45	18.14 ± 28.27	.385	21.61 ± 31.92	21.75 ± 31.60	21.46 ± 32.60	.919
Diarrhea	11.20 ± 20.69	12.51 ± 19.12	9.86 ± 22.32	.198	14.57 ± 19.65	14.04 ± 21.22	15.15 ± 18.02	.517
Financial difficulties	14.94 ± 26.12	14.78 ± 25.19	15.11 ± 27.33	.919	11.61 ± 20.82	11.50 ± 22.75	11.73 ± 18.75	.601

Risk group was defined separately for each disease: for PV, low risk (age ≤ 60 years and no history of thrombosis) versus high risk (age > 60 years or a history of thrombosis); for ET, the 4 R-IPSET categories were collapsed into low risk (very low + low) and high risk (intermediate + high) for comparison with the two-tier PV classification.

*P*-values were obtained using the Mann–Whitney *U* test (low vs high risk within each subtype).

ET = essential thrombocythemia, M = mean, PV = polycythemia vera, SD = standard deviation.

### 5.4. Factors affecting quality of life

Multiple regression analysis showed that global health status/QoL was significant (*F* = 30.54, *P* < .001). Significant factors were fatigue (β = −0.36, *P* < .001), numbness/tingling (β = −0.18, *P* = .030), disease duration (β = −0.17, *P* = .002), education of middle school or below (β = −0.13, *P* = .015), and weight loss (β = −0.12, *P* = .027). The functional scale was significant (*F* = 36.27, *P* < .001), with numbness/tingling (β = −0.30, *P* < .001), fatigue (β = −0.25, *P* < .001), bone pain (β = −0.22, *P* < .001), education of middle school or below (β = −0.17, *P* = .002), and fever (β = −0.12, *P* = .024) as significant factors. The symptom experience scale was significant (*F* = 39.27, *P* < .001), with fatigue (β = .28, *P* < .001), abdominal discomfort (β = .24, *P* < .001), bone pain (β = .20, *P* < .001), weight loss (β = .19, *P* < .001), fever (β = .18, *P* < .001), education of middle school or below (β = .16, *P* = .002), and female sex (β = .10, *P* = .041) as significant factors (Table 4).

**Table 4:** Factors affecting quality of life.

						N = 189
Variables	*B*	SE	β	*t*	95% CI	*P*
Global health status/QoL						
(Constant)						
Fatigue	−3.10	0.61	−0.36	−5.08	−4.31 to −1.90	<.001
Numbness/tingling	−1.45	0.66	−0.18	−2.18	−2.76 to −0.14	.030
Disease duration (yr)	−0.73	0.23	−0.17	−3.18	−1.18 to −0.28	.002
Education ≤ middle school (ref ≥ high)	−8.15	3.33	−0.13	−2.45	−14.73 to −1.58	.015
Weight loss	−1.47	0.66	−0.12	−2.24	−2.77 to −0.17	.027
*R^2^* = 0.54, Adjusted *R^2^* = 0.52, *F *= 30.54, *P *< .001, DW = 2.07						
Functional scales						
(Constant)						
Numbness/tingling	−1.33	0.32	−0.30	−4.14	−1.96 to −0.69	<.001
Fatigue	−1.17	0.34	−0.25	−3.42	−1.85 to −0.49	<.001
Bone pain	−0.95	0.26	−0.22	−3.71	−1.45 to −0.44	<.001
Education ≤ middle school (ref ≥ high)	−5.54	1.80	−0.17	−3.09	−9.08 to −2.00	.002
Fever	−1.42	0.63	−0.12	−2.27	−2.66 to −0.19	.024
*R^2^* = 0.50, Adjusted *R^2^* = 0.48, *F* = 36.27, *P *< .001, DW = 2.14						
Symptom scales						
(Constant)						
Fatigue	1.76	0.36	0.28	4.91	1.05 to 2.47	<.001
Abdominal discomfort	1.53	0.35	0.24	4.33	0.83 to 2.23	<.001
Bone pain	1.16	0.33	0.20	3.56	0.52 to 1.80	<.001
Weight loss	1.63	0.46	0.19	3.56	0.73 to 2.54	<.001
Fever (MPN-SAF item)	2.80	0.76	0.18	3.69	1.30 to 4.29	<.001
Education ≤ middle school (ref ≥ high)	7.11	2.26	0.16	3.14	2.64 to 11.57	.002
Female sex (ref male)	3.51	1.71	0.10	2.06	0.14 to 6.88	.041
*R^2^* = 0.60, Adjusted *R^2^* = 0.59, *F *= 39.27, *P *< .001, DW = 2.14						

β = standardized coefficient, *B* = unstandardized coefficient, CI = confidence interval, DW = Durbin–Watson, MPN-SAF = Myeloproliferative Neoplasm Symptom Assessment Form, SE = standard error.

## 6. Discussion

In this study, factors affecting the QoL of patients with PV or ET, including overall QoL, functional scales, and symptom experience, were identified as fatigue, disease duration, numbness/tingling, bone pain, fever, weight loss, abdominal discomfort, educational level, and female sex. The global health status/QoL scores were 61.06 and 61.10 out of 100 for patients with PV and ET, respectively; functional scale scores were 72.96 and 74.01, respectively; and symptom experience scores were 21.21 and 19.69, respectively. In comparison, QoL scores in Turkish patients with PV and ET were 66.7 and 75.0, with functional and symptom scale scores of 35.6 and 33.3, and 35.8 and 38.5, respectively.^[25]^ Although the overall QoL in our study population was lower than that in the Turkish study,^[25]^ functional scale scores were higher, and symptom scale scores were lower. This difference may be attributed to the inclusion of patients with disease duration exceeding 5 years in our study, whereas the Turkish study^[25]^ primarily included patients within 5 years post-diagnosis who were actively undergoing treatment.

In our study, 93.0~93.3% of patients experienced severe fatigue. In contrast, the prevalence of fatigue in the Turkish study ranged from 19.5% to 41.5%. Previous studies in Western populations^[27]^ have shown that fatigue is associated with tumor burden, weight loss, sleep and nutritional disturbances, treatment toxicity, pain, and anemia.^[29]^ Fatigue is also more common during treatment with cytoreductive agents, such as hydroxyurea, anagrelide, or interferon, particularly regimens including α-interferon.^[25]^ Fatigue is a subjective symptom that progressively worsens due to cancer or its treatment, negatively affecting physical, emotional, and cognitive aspects of QoL and interfering with daily functioning.^[30]^ Therefore, it is essential to identify contributing factors to fatigue and implement active interventions for affected patients.

Educational level was associated with QoL across all 3 domains, consistent with previous studies linking socioeconomic status to health outcomes in cancer populations.^[31,32]^ Lower educational attainment may limit access to health information, symptom self-management skills, and health-seeking behavior, thereby adversely affecting QoL. Although monthly income was not retained as a significant predictor in the final regression models, income remains a relevant contextual factor: in South Korea, where few treatments for PV and ET are covered by national health insurance,^[33]^ the financial burden may still affect patients’ access to care and warrants further investigation.

Disease duration and sex were also associated with QoL. Longer disease duration was associated with better global health status/QoL, in line with the more favorable functional and symptom scores observed in our cohort compared with the Turkish study,^[25]^ which enrolled patients earlier in the disease course. Female sex was associated with a greater symptom burden in the symptom scale model, similar to prior MPN cohorts reporting higher symptom severity among women.^[34]^

Symptoms related to myeloproliferative neoplasms, including numbness/tingling, bone pain, fever, weight loss, and abdominal discomfort, were also significant contributors to QoL. Numbness and tingling in PV and ET are thought to result from elevated blood viscosity and reduced peripheral circulation associated with erythrocytosis or thrombocytosis, which can also elevate thrombotic risk and necessitate continuous monitoring.^[2]^

Bone pain, caused by increased intramedullary pressure and inflammatory cytokines,^[35–37]^ limits daily activity and social participation.^[38,39]^ Patients may experience weight loss due to early satiety or abdominal discomfort, and fever due to inflammatory cytokine release.^[16,40]^ These diverse symptoms may also lead to emotional issues such as depression and anxiety.^[41]^ As there is no curative treatment for PV or ET, therapeutic goals focus on controlling blood counts, managing disease-related symptoms including splenomegaly, reducing the risk of thrombo-hemorrhagic complications, and delaying disease progression to post-PV/ET myelofibrosis or acute leukemia.^[42]^

Thrombosis risk stratification did not distinguish overall symptom burden or QoL between low- and high-risk patients within either PV or ET. Among individual items, only weight loss (both PV and ET), abdominal discomfort, appetite loss, and emotional functioning (ET only) differed significantly by risk group. This pattern is broadly similar to the MPN Landmark survey, in which prognostic risk score was not consistently correlated with QoL, and a majority of respondents with low calculated risk scores still reported that their symptoms reduced QoL (PV, 62%; ET, 57%).^[18]^ These findings suggest that symptom burden and QoL are not fully captured by thrombosis risk category alone and warrant further investigation in low-risk patients.

In this study, the mean MPN-SAF TSS scores were 22.12 and 20.73 for PV and ET, respectively, with fatigue occurring in 93.3% and 93.0% of patients. In comparison, US patients with PV and ET had mean scores of 17.4 and 14.8, and the prevalence of fatigue and early satiety was 73% and 71%, respectively.^[18]^ Although the overall phlebotomy rate was 40.2% (76 of 189 patients), phlebotomy is rarely indicated in ET; among patients with PV, 76 of 87 (87.4%) underwent phlebotomy, comparable to the 90% reported in US cohorts. Therefore, phlebotomy use alone is unlikely to explain the higher symptom burden observed in our sample. Broader structural factors, including delayed diagnosis, limited treatment options, and lack of proactive intervention in Korea,^[43,44]^ together with limited insurance coverage for newer cytoreductive agents,^[33]^ may better account for this difference. Further studies directly comparing treatment access and healthcare delivery between countries are needed to clarify these findings.

Using instruments such as MPN-SAF TSS and EORTC QLQ-C30 is important for the longitudinal assessment of patient status during treatment.^[20]^ Repeated evaluation allows for effective symptom management and determination of intervention strategies. Additionally, it facilitates a detailed understanding of patient status by healthcare providers, improves communication between patients and providers, enhances patient awareness and self-management, and enables nurses to identify and address patient needs.^[16,18]^ Overall, these findings indicate that quality of life in patients with PV and ET is shaped by a complex interplay of symptom burden, socioeconomic factors, and clinical characteristics, highlighting the need for integrated, patient-centered approaches to long-term disease management.

## 7. Conclusions and clinical implications

This study aimed to identify factors affecting the QoL of patients with PV and ET by assessing symptom burden and QoL using the EORTC QLQ-C30 and MPN-SAF TSS. The results indicated that fatigue, disease duration, numbness/tingling, bone pain, fever, weight loss, abdominal discomfort, educational level, and female sex significantly influenced QoL. These findings suggest the importance of active symptom management and continuous monitoring. However, as this study was conducted in a single institution and did not assess comorbidities, future research should evaluate how comorbid conditions affect symptom burden and QoL in patients with PV and ET. These findings underscore the need for psychosocial providers and multidisciplinary care teams to routinely assess symptom burden and quality of life in patients with PV and ET and support the development of policies that integrate systematic symptom monitoring and psychosocial support into standard care.

## Author contributions

**Conceptualization:** Yoorin Cho.

**Data curation:** Yoorin Cho.

**Formal analysis:** Yoorin Cho.

**Investigation:** Yoorin Cho.

**Methodology:** Yoorin Cho.

**Project administration:** Yoorin Cho.

**Validation:** Yoorin Cho.

**Writing – original draft:** Yoorin Cho.

**Writing – review & editing:** Yoorin Cho.
